# Non-contact optical in-vivo sensing of cilia motion by analyzing speckle patterns

**DOI:** 10.1038/s41598-022-20557-1

**Published:** 2022-10-05

**Authors:** Doron Duadi, Nadav Shabairou, Adi Primov-Fever, Zeev Zalevsky

**Affiliations:** 1 Faculty of Engineering and Institute of Nanotechnology and Advanced Materials, Bar Ilan University, 5290002 Ramat Gan, Israel; 2 Department of Otolaryngology and Head and Neck Surgery, Sheba Medical Center, and Sackler School of Medicine, Tel-Aviv University, Tel-Aviv, Israel

**Keywords:** Medical research, Optics and photonics

## Abstract

Cilia motion is an indicator of pathological-ciliary function, however current diagnosis relies on biopsies. In this paper, we propose an innovative approach for sensing cilia motility. We present an endoscopic configuration for measuring the motion frequency of cilia in the nasal cavity. The technique is based on temporal tracking of the reflected spatial distribution of defocused speckle patterns while illuminating the cilia with a laser. The setup splits the optical signal into two channels; One imaging channel is for the visualization of the physician and another is, defocusing channel, to capture the speckles. We present in-vivo measurements from healthy subjects undergoing endoscopic examination. We found an average motion frequency of around 7.3 Hz and 9.8 Hz in the antero-posterior nasal mucus (an area rich in cilia), which matches the normal cilia range of 7–16 Hz. Quantitative and precise measurements of cilia vibration will optimize the diagnosis and treatment of pathological-ciliary function. This method is simple, minimally invasive, inexpensive, and promising to distinguish between normal and ciliary dysfunction.

## Introduction

Biophotonics is a science that uses tissue optics as a basis for designing innovative optical diagnostic and treatment technologies^1^. There are advanced methods for tissue evaluation that use the properties of light to enable the clinician to make an instant diagnosis, which was previously possible only by using histological analysis. Optical systems based on light-tissue interaction have great potential to improve endoscopic diagnosis^2^.

Over the last few years, improvements in endoscopic imaging technologies have enabled the identification of early cancers, delicate structural patterns of the mucosa, and visualization of abnormal patterns from the mucosal surface. Presently, white light endoscopy is the primary method used for wide-area imaging in the medical field. Endoscopic imaging techniques available in routine practice include chromoendoscopy^3^, confocal microscopy^4^, optical coherence tomography^5^, autofluorescence imaging^6^, and narrow-band imaging^7^. They have the capability of imaging wide areas in real-time and specifically focusing on abnormal targeted areas. These methods improve the image resolution, contrast, and tissue penetration, and provide biochemical and molecular information about different diseases on the tissue surface. These techniques are readily available and simpler, yet they are time-consuming and require special training. Hence, those methods are unlikely to replace conventional biopsy with a histopathologic interpretation of excised tissue any time soon. Rather, they are more likely to provide a more accurate and efficient approach to target biopsy of diseased tissue^8^.

Due to these progressions, we propose an innovative and applicable technique of non-contact in-vivo measurement of cilia motion through an endoscope.

Cilia are hair-like structures that exist on the surface of cells. Ciliated epithelial cells are found along the upper and lower respiratory tract. Each cell has hundreds of motile cilia^9^. The activity and movement of the cilia are an essential defense mechanism of the airway due to mucus transport. The ciliated epithelial cells are lined with two layers of surface liquid: The periciliary layer is of low viscosity and approximates the height of the cilia. This layer provides optimal surroundings for the ciliary beating. The mucus layer is on top of the periciliary layer. It is composed of the secretions of the goblet cells and the submucosal glands. The mucus layer is a heterogenous, adhesive, viscoelastic gel. The mucus layer traps foreign particles such as dust, toxic substances, bacteria, viruses, and allergens inhaled from the nose^10,11^. The coordinated beating of the ciliated epithelial cells transports the mucus to the pharynx. The mucus is then swallowed or coughed. The permanent clearance of the mucus is an essential defense mechanism of the respiratory tract. Each ciliated cell has about 200 motile cilia. The cilia are a protrusion of about 6 µm from the cell's surface. Cilia beat in a coordinated fashion in normal airways, at a rapid frequency of 7–16 Hz. The coordinated beating of the cilia mobilizes the mucous on top. Disorders of the conciliar transport can be caused by ciliary dysfunction, as in Primary Ciliary Dyskinesia (PCD), or by increased viscosity of the respiratory secretions as in Cystic fibrosis (CF); other causes for conciliary dysfunctions are infection, inflammation, and exposure to ciliotoxicity agents^12,13^.

The mucociliary transport along the respiratory tract can be measured by several in-vivo and in-vitro techniques^14^. There are two types of in-vivo transport measurements: the saccharine test and the nuclear test. In the saccharine test, a particle of saccharine colored with methylene blue is placed on the inferior turbinate of the nose. The time it takes the patient to taste saccharine and until the blue color of the particle appears in the pharynx should be less than 30 min. The Nuclear test uses a minute amount of Tc99m radiolabeled albumin placed on the inferior turbinate or the nasal septum. A γ-camera follows the migration of the radioactivity that normally should disappear from the nasal cavity within 30 min.

*In-vitro* transport measurements use contrast microscopy to check the presence of cilia in brushed or bioptic materials. Displacement and rotations of cell clusters or cell sheets within the fluid and movement of particles within the fluid lining the cilia are criteria for the presence of coordinated activity. The absence of this movement in the presence of ciliary activity is determined as "uncoordinated ciliary activity". With high-speed camera (< 500 Hz) microscopy analysis, it is possible to examine the ciliary beat cycles, beat pattern, amplitude, degree, and speed. However, it is not yet sufficiently standardized^15–17^.

Optical coherence tomography (OCT) allows visualization of ciliated epithelium^18–20^. However, the resolution of the OCT system is not high enough to observe the actively beating cilia. To enhance the contrast of visualization of cilia motion speckle variance OCT (SV-OCT) can be used. Moreover, for the detection of cilia flow speed, a particle tracking velocimetry OCT (PTV-OCT) is suitable. The combination between speckle variance imaging and PTV-OCT analysis required high computational power and time, which does not allow real-time analysis^21^. In addition, sensing of ciliary beat frequency by Doppler OCT (D-OCT) can measure vibrations through interferometry in living tissues but is an expensive and complex technique^22^.

Our proposed optical technique is based on tracking temporal changes of the spatial distribution of defocused speckle patterns. The speckle patterns are created by the backscattered light from the surface roughness of the inspected object^23^. Each speckle pattern is a self-interfered pattern that serves as a reference point that tracks the changes in the light phases while being scattered from the object’s surface. This technique was previously demonstrated for non-contact measuring of biomedical parameters through external configuration, such as monitoring heart rate, blood pulse pressure^24,25^, glucose concentration in blood^26,27^, bone fractures^28^, melanoma^29^, and through internal configuration for middle ear effusion detection^30^ and measuring vocal folds vibration^31^.

In this paper, we use an endoscopic configuration to perform an in-vivo measurement of the motion frequency of cilia in the nasal cavity of healthy subjects. The optical setup enables two measurements simultaneously: white light imaging of the nasal cavity and defocused measurements of the speckle pattern. We take a video of the speckle patterns due to cilia motion. Then we calculate the correlation between the frames and analyze the spectrum.

Our method is in-vivo, simple to perform, minimally invasive, cheap, reproducible, and accurate to examine the motility of the cilia in the nasal cavity and differentiate between normal and pathological-ciliary function. We will show that when the endoscope is directed to the mucosa covering the nasal cavity, there is a spectral peak around 7.3 Hz and 9.7 Hz. However, no spectral peak can be detected when the endoscope is not directed to the epithelial ciliated mucosa.

Note that this research is a proof of concept that there is an ability to measure the frequency of the movement of the cilia in the nasal cavity using the speckle-based endoscopic method. The next research will be done on patients and will demonstrate the ability to distinguish between healthy subjects and pathological subjects.

## Methods

### Experimental setup

**Figure 1:**
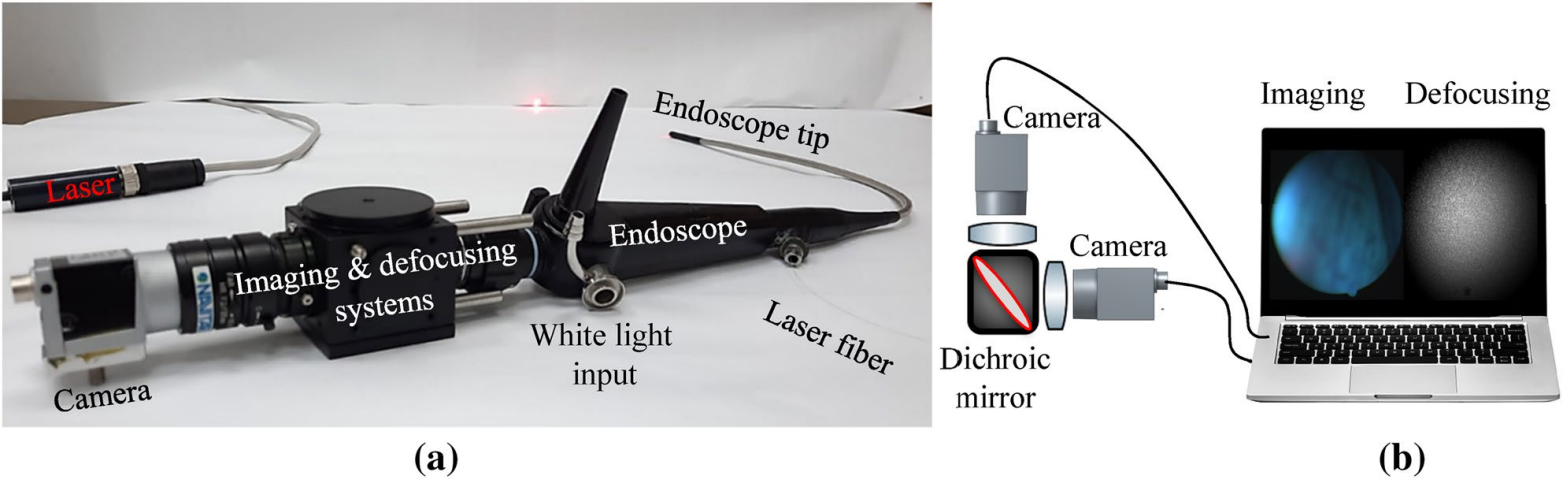
The optical setup for cilia motion characterization. (**a**) The setup consisted of an endoscope, a 650 nm fiber laser, an imaging system, and a defocusing system for speckle patterns. (**b**) The imaging system and the defocusing system consisted of lenses and cameras connected to a black box with a 650 nm dichroic mirror.

The optical setup (Fig. 1a) includes a laser projected through a flexible endoscope (Therapeutic PENTAX FNL-15RP3) used to view the subject’s nasal cavity cilia. Endoscopes are commonly used in medical procedures at the ear, nose, and throat (ENT) clinic (according to section “Human measurements”). The endoscope had a 2.1 mm diameter instrument channel, a 4.9 mm diameter insertion tube, and a view angle of $75^\circ$ (with a 0.6 NA). A fiber was inserted into the endoscope's working channel and the tip of the endoscope was directed to the nasal cavity. The laser (FP-FCL-660-30MD-800-FC, LASER COMPONENTS, Germany), collimated through the optical fiber, had a 650 nm wavelength, with a 5mW output power. The laser source wavelength was 650 nm which has low absorption in human tissue and allows using a weak biocompatible laser source with low power. In order to obtain a single point of illumination, a GRIN lens (GRIN2906, Thorlabs, US) was attached to the end of the fiber.

The configuration consisted of two optical systems, both had lenses and cameras connected to the computer. One to capture the speckle pattern and the second for visualization of the area of interest. The systems were connected to a black plastic box coupled to the endoscope output (imaging & defocusing system in Fig. 1a). The box split the backscattered light by a 650 nm dichroic mirror into speckle sensing and visual imaging (to locate the beam at the cilia area) simultaneously (Fig. 1b).

The defocusing system extracted the movement of the cilia by correlating the reflected speckle patterns and analyzing the temporal changes in the location of the correlation peak. The speckle patterns were created by illuminating the mucosa of the nasal cavity. To sense the cilia vibrations, the camera (Basler acA800-510um) was defocused to the far-field region, connected to a lens with a focal length of 70 mm. The lens generated a defocused image of speckles on the camera’s detection array at the rate of 200 frames per second (fps). The second imaging system allowed the clinician to visualize the illuminated location of the nasal mucosa. This system consisted of a lens with a focal length of 25 mm that was attached to the camera (Basler acA800-510uc). In addition, the endoscope was connected to a white light source which enable the doctor to watch the video on our computer and to identify that the tip of the endoscope was directed to the mucosa. The camera captured the images at 200fps, with a pixel size of 4.8 µm. Our field of view (FOV) is 512 × 512 pixels, which means that under an endoscope magnification with a factor of X3, the FOV is 7.4 × 7.4 mm. While the physician looks at the full white field FOV, we only analyzed the region in the image containing speckle patterns. Hence, the size of the analyzed area is only 1.2 × 1.2 mm, which is approximately the laser beam diameter.

The temporal resolution depends on the equation:

1 $$\Delta t = \frac{1}{{Frame\;{ }Rate}},$$

while the frequency resolution depends on the equation:

2 $$\Delta f = \frac{{Frame\;{ }Rate}}{N},$$

where N is the number of frames recorded and the frame rate is a camera-dependent parameter. Hence, the system detection limit depends on the frame rate of the camera and the number of frames.

### Analysis of temporal-spectral information

The analysis was based on temporal tracking of back-reflected speckle patterns generated while illuminating the inspected object with a laser beam^32^. This method allows the monitoring of nanometric vibrations. In the configuration, the detection of vibration frequency was done by analyzing speckle patterns. The reflected light was collected only by a lens and fast imaging camera while the epithelial ciliated mucosa was illuminated with a laser. As previously mentioned, the speckle patterns were collected through the optics in which the object itself was defocused, therefore the camera was focused on the far-field^33^.

In the far-field, tilting-related vibrations cause linear movement, rather than changes, of the speckle patterns. It means the tilting movement which generates a linearly space-dependent phase change, is translated to movement at the transversal plane (X and Y plane at the far-field), proportionally to a tilt movement of the inspected surface. Therefore, it is expressed by the Fourier transform as a lateral shift of the speckle patterns. It allows measuring the object's displacement by tracking the temporal change of the correlation maxima^34,35^.

**Figure 2:**
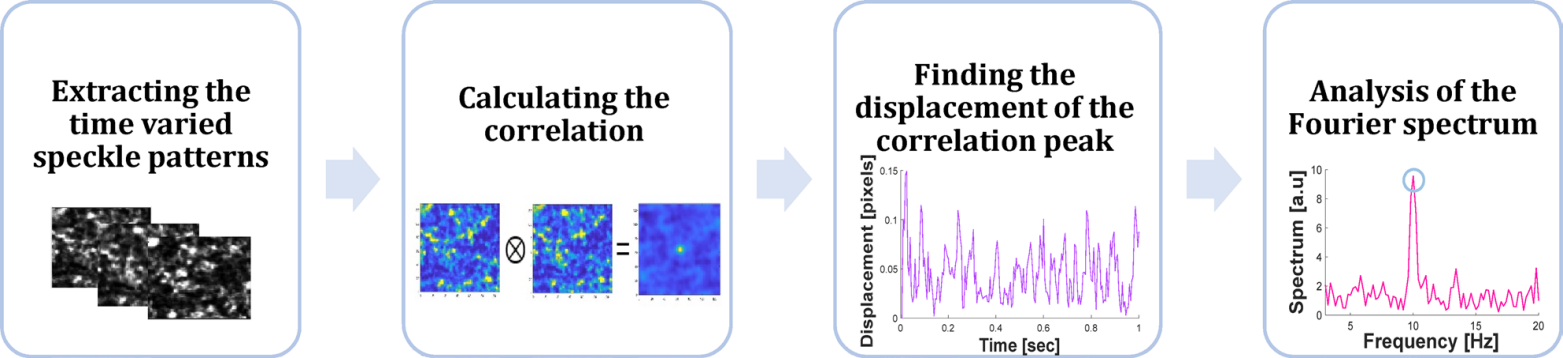
Flow chart of the image processing algorithm for monitoring cilia motion frequency identification.

Thus, the speckle pattern of each frame was shifting due to the cilia movement. The processing of the captured images (Fig. 2) included calculating the correlation between a given frame ($I_{n} \left( {x,y} \right)$) and the first frame ($I_{1} \left( {x,y} \right)$) of the time-varied speckle patterns:

3 $$C_{n} \left( {x,y} \right) = \smallint I_{n} \left( {x + u,y + v} \right) \cdot I_{1} \left( {u,v} \right)dudv.$$

For each two frames n and the first frame, the location of the maximum of $C_{n}$ is found, denoted as $r_{n}$. The change in the location of the correlation peak ($r_{n}$) in time was extracted ($r\left( t \right) = \left\{ {r_{n} } \right\}$). Since the cilia motion changes the speckle pattern observed by the camera, there is a change in the location of the correlation peak. Analysis of the time dependency of the correlation peak's position indicates the corresponding tilting of the cilia. The movement frequency is identified in the Fourier spectrum.

4 $$R\left( f \right) = FFT\left\{ {r\left( t \right)} \right\}$$

where $FFT$ denotes the Fourier transform. The cilia motion frequency is the maximum value in the Fourier transform between 7 and 16 Hz.

The image processing of the speckle temporal movement was analyzed by MATLAB. The workflow indicates that information can be recorded and processed within a reasonable amount of time to generate information about the cilia beat. Fourier transforms are not normally computationally intensive and can be done in real-time.

**Figure 3:**
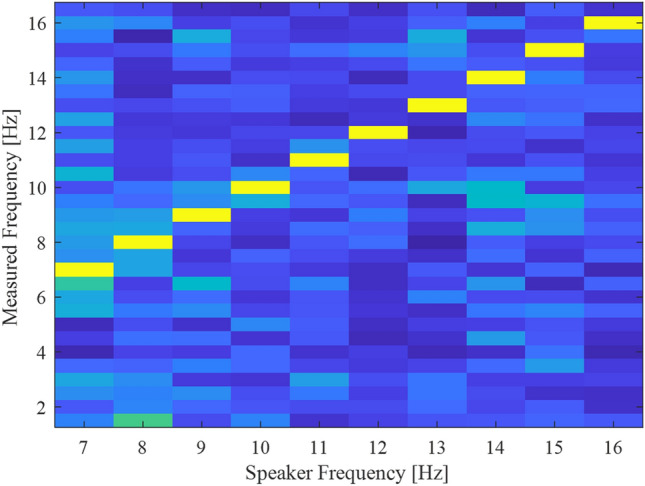
System calibration using a speaker. We used a speaker to create vibrations in a frequency range of 7–16 Hz and measured the spectrum with our system. The system detected the correct peak frequency with a 0.5 Hz accuracy.

In order to validate the system's ability to measure in the frequency range of 7–16 Hz, we used a speaker to transmit a known frequency and measured it through the endoscope. The camera captured the images at 200 fps for 2 s, thus the number of frames was 400. Hence, the frequency resolution was ± 0.5 Hz. The spectrum of the measured frequency is presented as a function of the speaker frequency (Fig. 3). One can see that there is a strong fit between the peak in the measured spectrum and the transmitted one (presented as a yellow diagonal line in the figure).

### Human measurements

In order to inspect the nasal cavity, the physician performs nasopharyngoscopy (nasopharynx-endoscopy). Nasopharyngoscopy is a diagnostic procedure that examines the nose and throat's internal structures and detects abnormalities in the nasopharyngeal area. The examination is performed by an ENT specialist and usually takes 2–4 min. During the procedure, the patient sits up straight in a chair. Local anesthesia can be used to minimize discomfort during the procedure. In our research, the doctor inserted cotton pads soaked in a solution of 2% Amethocain through the nostrils for 5 min without the use of decongestants.

The density of ciliated cells increases in the nasal cavity, in the antero-posterior direction, and in the nasal sinuses^36^. Hence, the recordings and measurements were performed about 3 cm deep into the nasal cavity before reaching the inlet of the nasopharynx. The measurements were performed from different spatial areas in the nasal cavity-multiple areas of ciliated mucosa (22 measurements) and the lumen of the nasal cavity (36 measurements), from several healthy subjects. The subjects were young healthy males between the ages of 20–30, without underlying diseases.

All research procedures were performed with approval by the Sheba IRB-Helsinki Committee (Institutional Review Board for human and animal trials). All experiments were performed in accordance with relevant guidelines and regulations, and informed consent was obtained from all subjects who participated in the research experiment.

## Results and discussion

Our endoscopic configuration (from section. “Experimental setup”) was used to examine the nasal cavity of healthy subjects. The laser illumination was pointed at various points of the nasal cavity area. The insertion of the laser fiber through the endoscope into the nasal cavity combined with the white light source provided the clinician a clear image of the nasal cavity and the ability to adequately direct the endoscope to the mucosa overlying the nasal cavity or not directly to the mucosa.

We illuminated different spatial areas in the nasal cavity (tenths of areas), then we compared their spectral behavior. The analysis was addressed for each speckle pattern created from the illumination point. The measurement analysis was performed based on speckle patterns analysis and the temporal spectrum of the specific region with laser illumination (according to section“ Analysis of temporal-spectral information”). The correlation displacement when the illumination was not directed to the nasal mucosa was our control. In addition, we illuminated the ciliated mucosa area.

**Figure 4:**
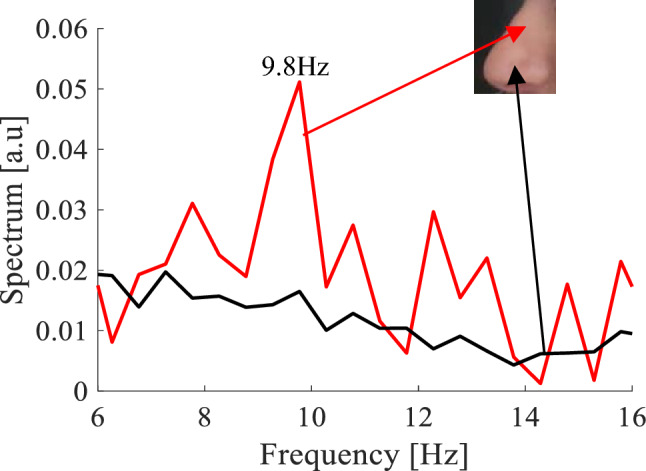
Detection of cilia vibration using the endoscopic configuration. Images were taken through an endoscope with laser illumination. The correlation spectrum extracted from the analysis of the speckle patterns (**a**) from the ciliated mucosa area, presents a peak in the spectrum at a frequency around 9.8 Hz, and (**b**) in the lumen of the nasal cavity no apparent peak exists.

When the tip of the endoscope was directed towards the lumen of the nasal cavity, which was our control area (black line in Fig. 4), we observed that the vibration frequency was very negligible. When the endoscope was directed laterally, to the epithelial ciliated mucosa, we observed in the illuminated area that the vibration frequency was 9.8 ± 0.5 Hz (red line in Fig. 4).

**Figure 5:**
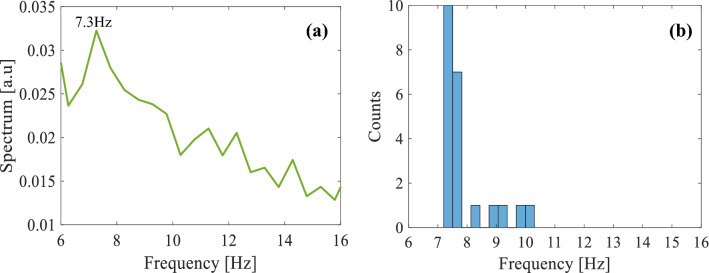
(**a**) The average spectrum of the measurements extracted from the mucosa at the nasal cavity presents a peak in the spectrum around 7.3 Hz and (**b**) a histogram of the motion frequencies extracted from multiple ciliated mucosa areas.

Next, some measurements of areas of the nasal mucosa were analyzed under laser illumination. We calculated the average spectrum of all the illuminated mucosal areas and found a peak at 7.3 ± 0.5 Hz (Fig. 5a). This was expected since the cilia beat at a frequency of 7–16 Hz, and the patient did not present any pathologies. Note, in voice signals additional peaks that appear in the frequency data have significance. However, for the detection of cilia motion, we look for the maximum peak frequency. Please note that there are blood vessels in the nasal cavity that flow at a frequency of 0.5–3 Hz^37^, therefore the frequency of the measurements we received is a result of the motion of the ciliated mucosa. Moreover, in the case of temporary data, the noise is due to the motion of the measurements, which is at low frequencies below 1 Hz. In addition, we presented a histogram of the different frequencies that were extracted from multiple ciliated mucosa areas (Fig. 5b).

According to section “Analysis of temporal-spectral information”, please note that we use a defocused lens to analyze the speckle pattern. This defocusing causes a loss of spatial resolution (between the measured vibration signal and the spatial domain). However, the defocusing is not very strong and still has a sufficient spatial resolution with a proper spatial allocation of the measured signal. In addition, in order to observe speckles, the source should be coherent with a long coherence length (about 1 cm). The laser source wavelength influences the penetration depth and the absorption in human tissue. Higher wavelengths lead to a high penetration depth and low absorption in the tissue.

## Conclusions

In this paper, the usage of non-contact optical configuration for the detection of cilia motility was presented. We proposed an innovative technique that will benefit the identification and diagnosis of pathological-ciliary function based on examination of the motion of the cilia in the nasal cavity, which allows improving the medical treatment. Our approach serves as an "optics biopsy" method for quantitative in-vivo estimation of cilia frequency movement. The proposed configuration consists of two cameras, two lenses, a dichroic mirror, a laser, and an endoscopic examination. We used this method to characterize the cilia motion of healthy patients undergoing endoscopy of the nasal cavity. During the examination, the endoscope was connected to a white light source and a camera. The camera was connected to a monitor to view the captured magnified images. In this way, it is possible to identify the nasal cavity and nasopharynx abnormalities. The endoscope we used in this research is intended for the ear, nose, and throat. However, using a different endoscope would allow examining different body cavities.

According to the medical literature, normally cilia have a beating motion of 7–16 Hz while moving constantly and synchronously. Therefore, the measurement of ciliary dynamics can serve as an important indicator of upper respiratory diseases. Detection of unsynchronized vibrations of the cilia means that the cilia mobility is damaged. Our method considers in-vivo measurements and allows directly sensing of the cilia vibration. Those measurements are much more accurate because they take into account the mucosal composition and environmental differences, which affect the cilia beat frequency.

The significant advantage of this approach is the ability to directly sense the cilia motion in the nasal cavity with a minimally invasive procedure and to analyze the existing temporal frequency. Analyzing these spectral responses is the first step toward a simple non-contact detection of cilia abnormalities. The next step is to examine subjects with PCD or CF. This enables an early, simple, and inexpensive diagnosis of pathological-ciliary dysfunction. It may help the clinician to provide faster treatment that will slow the progression of the diseases. In addition, it can contribute to the understanding of cilia motion characteristics.

## Data Availability

The datasets generated during and/or analyzed during the current study are not publicly available due to patient privacy concerns but are available from the corresponding author on reasonable request.
